# Supplementary material for: NKG2A is a NK cell exhaustion checkpoint for HCV persistence
Source: Nat Commun. 2019 Apr 3;10:1507. doi: 10.1038/s41467-019-09212-y (PMC6447531; doi:10.1038/s41467-019-09212-y)
Supplement: Supplementary file 1 — Supplementary Information [file 41467_2019_9212_MOESM1_ESM.pdf]

Supplementary Information for

NKG2A is a NK cell exhaustion checkpoint for HCV persistence

Zhang et al.

This PDF file includes:

Supplementary Figure 1 to 10 and Legends

Supplementary Table 1 and 2

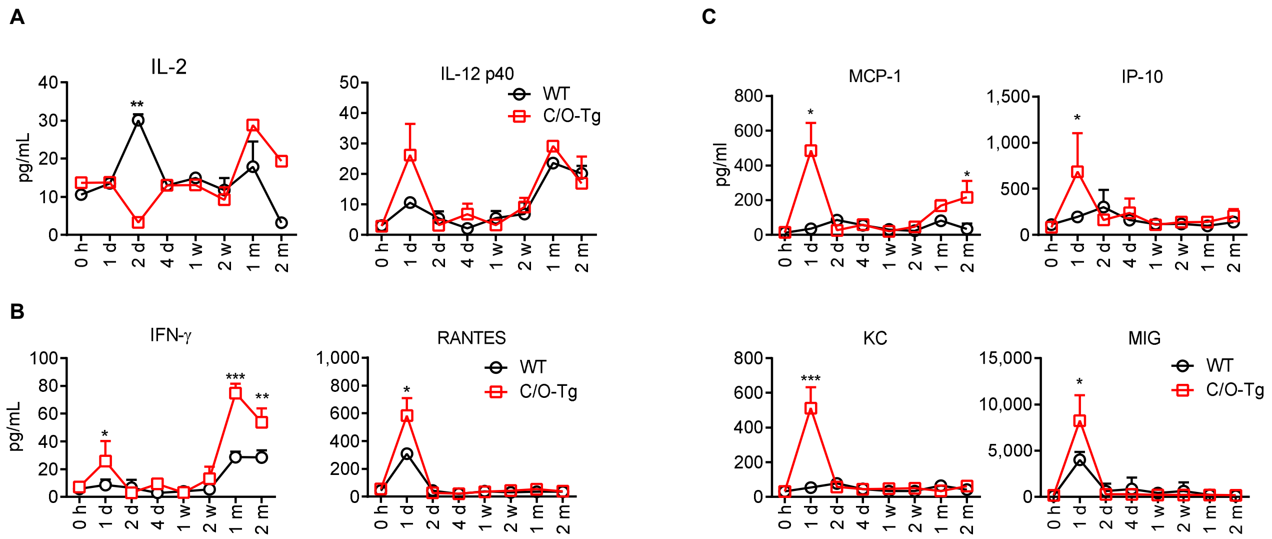

Supplementary Figure 1. Dynamics of serum chemokines and cytokines after J339EM infection.
Serum levels of Th1 cytokines (**A**), proinflammatory cytokines (**B**) and chemokines (**C**) were detected by LUMINEX assay. Other cytokines and chemokines (IL-3, IL-4 IL-5, IL-15, IL-12p70, IL-21, IL-23, IL-33, G-CSF, Eotaxin, TNF-α) without any difference between wt and transgenic mice were not shown. Data were mean ± SD, ANOVA test. *, $P < 0.05$; **, $P < 0.01$; ***, $P < 0.001$. Source data are provided as a Source Data file.

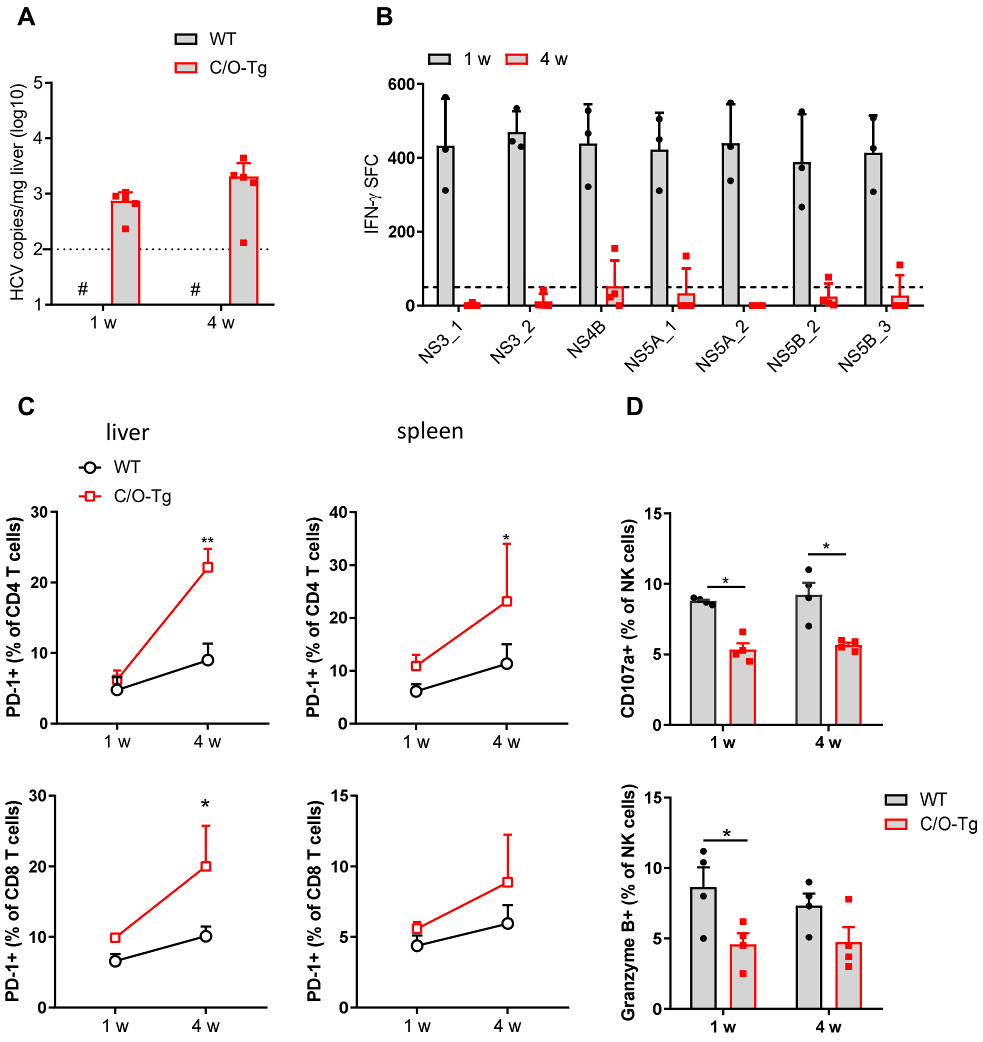

Supplementary Figure 2. Mice infected with patient sera positive for HCV1b.
C/O^{Tg} or wt mice (n = 5 for each time point) were *i.v.* infected with 200 uL HCV1b patient sera (3.93 x 10^6 copies/mL). (**A**) HCV genome copies in liver. (**B**) HCV specific $CD8^+$ T cells response, and (**C**) PD-1 expression in $CD4^+$ and $CD8^+$ T cells isolated from spleens or livers. (**D**) NK cells were evaluated by FACS analysis. Data were mean $\pm$ SD, student *t* test. *, $P < 0.05$; **, $P < 0.01$. Source data are provided as a Source Data file.

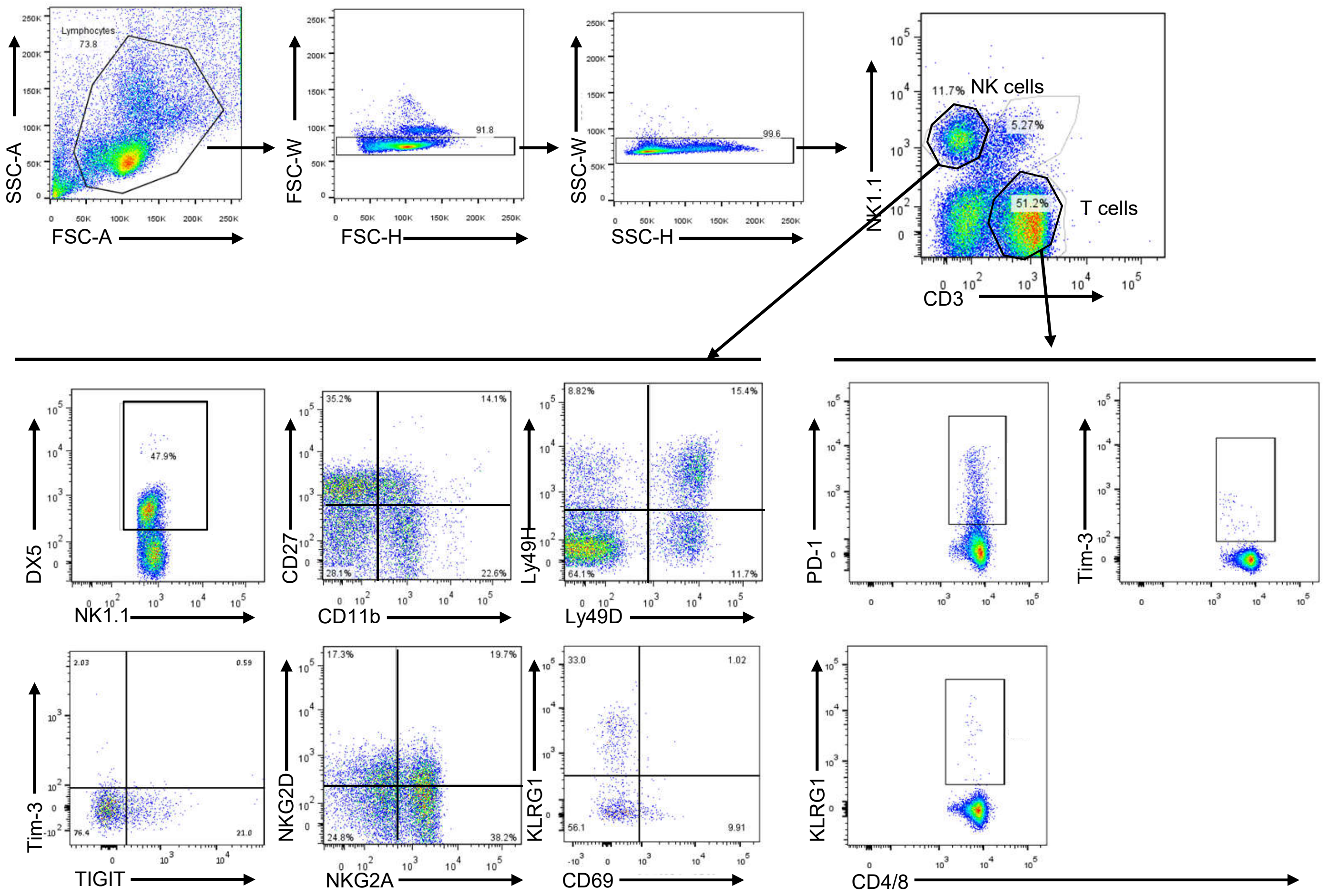

Supplementary Figure 3. Gating strategies for flow cytometric analysis.
NK cells were gated as $CD3^{+}$ $NK1.1^{+}$ and analyzed for the expression of indicated markers. $CD4^{+}$ T and $CD8^{+}$ T cells were gated and analyzed for the expression of inhibitory markers.

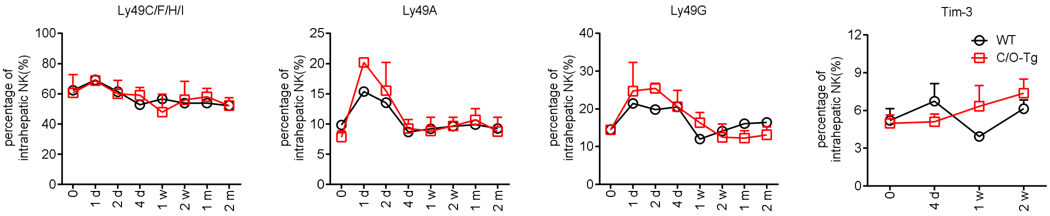

Supplementary Figure 4. Expression of inhibitory Ly49 family receptors and Tim-3 on hepatic NK cells in HCV infected mice.
Mice were treated as in Figure 1A. Analysis of indicated NK cell surface markers by FACS. Data were mean ± SD. Source data are provided as a Source Data file.

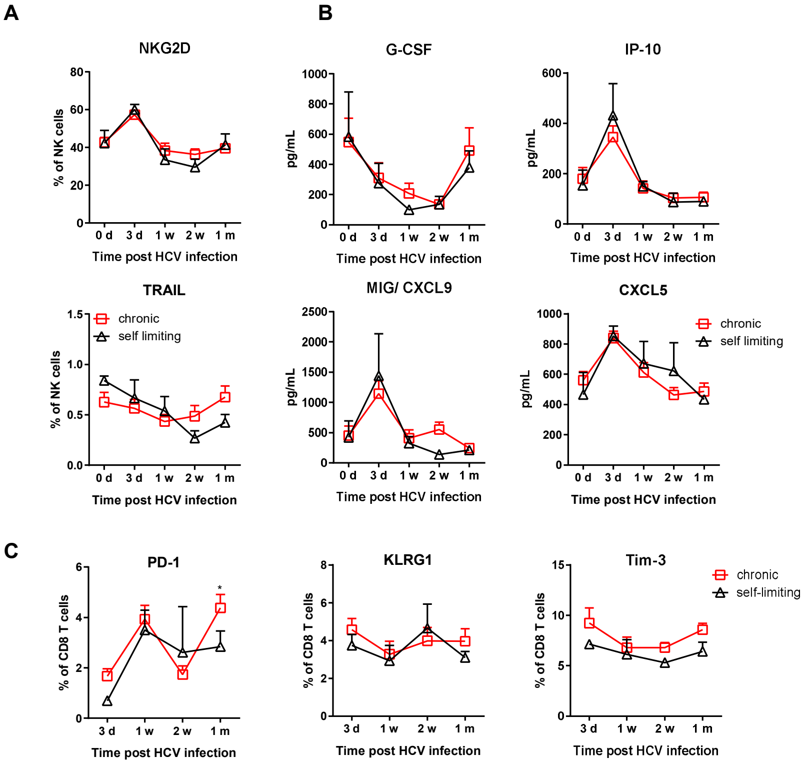

Supplementary Figure 5. Candidate factors to discriminate self-limiting from chronic infections in HCV infected mice.
Mice were treated as in Figure 3A. (**A**) Analysis of NK cell surface markers by FACS. (**B**) Analysis of serum cytokines and chemokines by LUMINEX assay. (**C**) Analysis of T cell inhibitory markers by FACS. Data were mean ± SD. ANOVA test. *, $P < 0.05$. Source data are provided as a Source Data file.

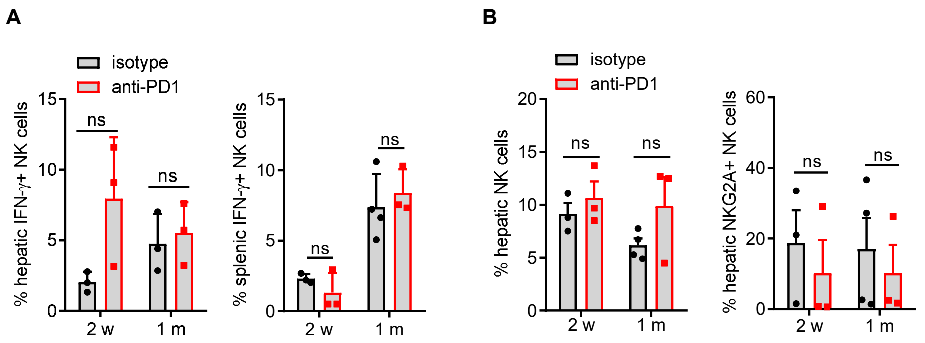

Supplementary Figure 6. PD-1 blockade alone was not able to restore NK cell function.
(**A-B**) Mice were treated as in Figure 1E, NK percentage, NKG2A+ and IFN-γ+ cells among NK cells were detected by FACS. Data were mean ± SD, ns, not significant. Source data are provided as a Source Data file.

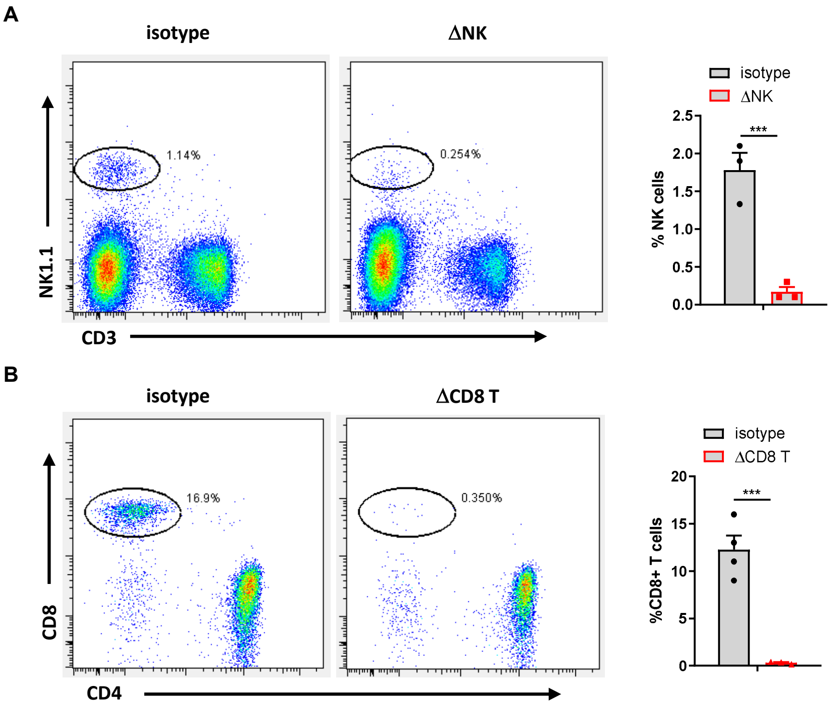

Supplementary Figure 7. Depletion efficiency of NK cells and CD8⁺ T cells.
Mice were treated as in Figure 4C. (**A**) Depletion efficiency of NK cells by anti-AGM1 examined by FACS in blood. (**B**) Depletion efficiency of CD8⁺ T cells by antibodies (TIB210) examined by FACS in blood. Data were mean ± SD, student *t* test. ***, P < 0.001. Source data are provided as a Source Data file.

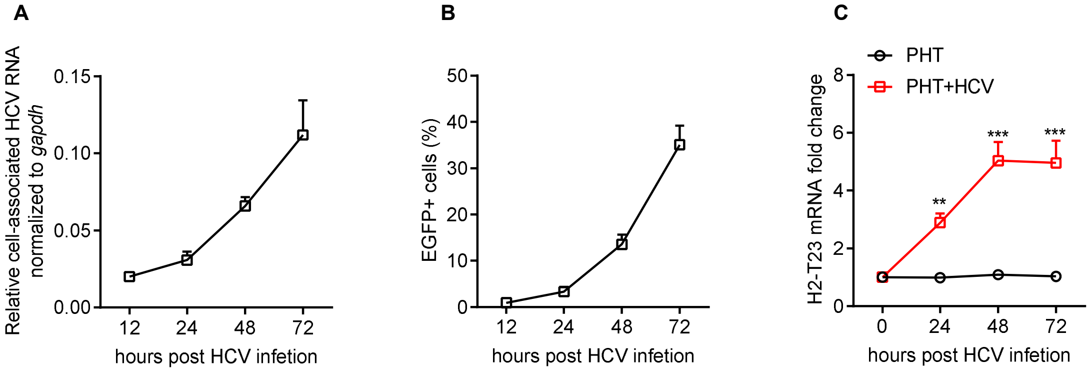

Supplementary Figure 8. HCV replication in C/O^{Tg} PHT and induction of Qa-1 upon infection.
C/O^{Tg} PHT were infected with HCV (J399EM) as in Figure 5A. (**A**) Dynamics of cell associated HCV RNA. (**B**) Percentage of EGFP positive cells after J399EM infection. (**C**) Qa-1 mRNA level after J399EM infection. Data were mean $\pm$ SD, student *t* test. **, $P < 0.01$. ***, $P < 0.001$. Source data are provided as a Source Data file.

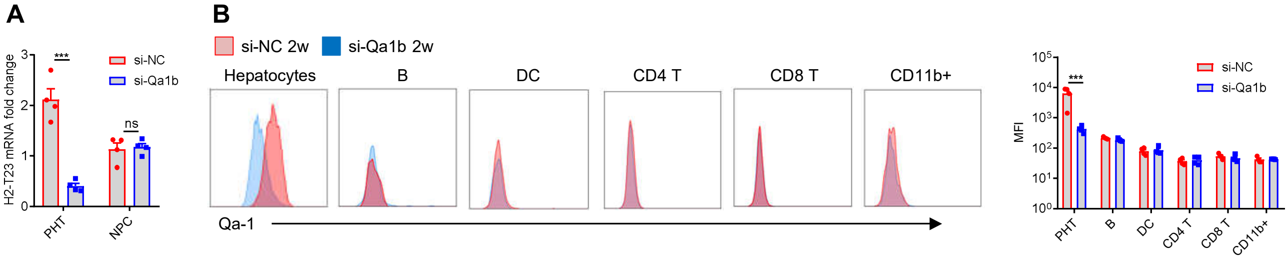

Supplementary Figure 9. Delivery of cholesterol conjugated siRNA by tail vein injection to knockdown Qa-1 expression in hepatocytes.
Mice were treated as in Figure 6G. Measurement of Qa-1 mRNA (**A**) and protein (**B**) level on hepatocytes or NPC subsets as indicated 2 weeks post HCV infection. Data were mean ± SD, student *t* test. ***, $P < 0.001$. ns, not significant. Source data are provided as a Source Data file.

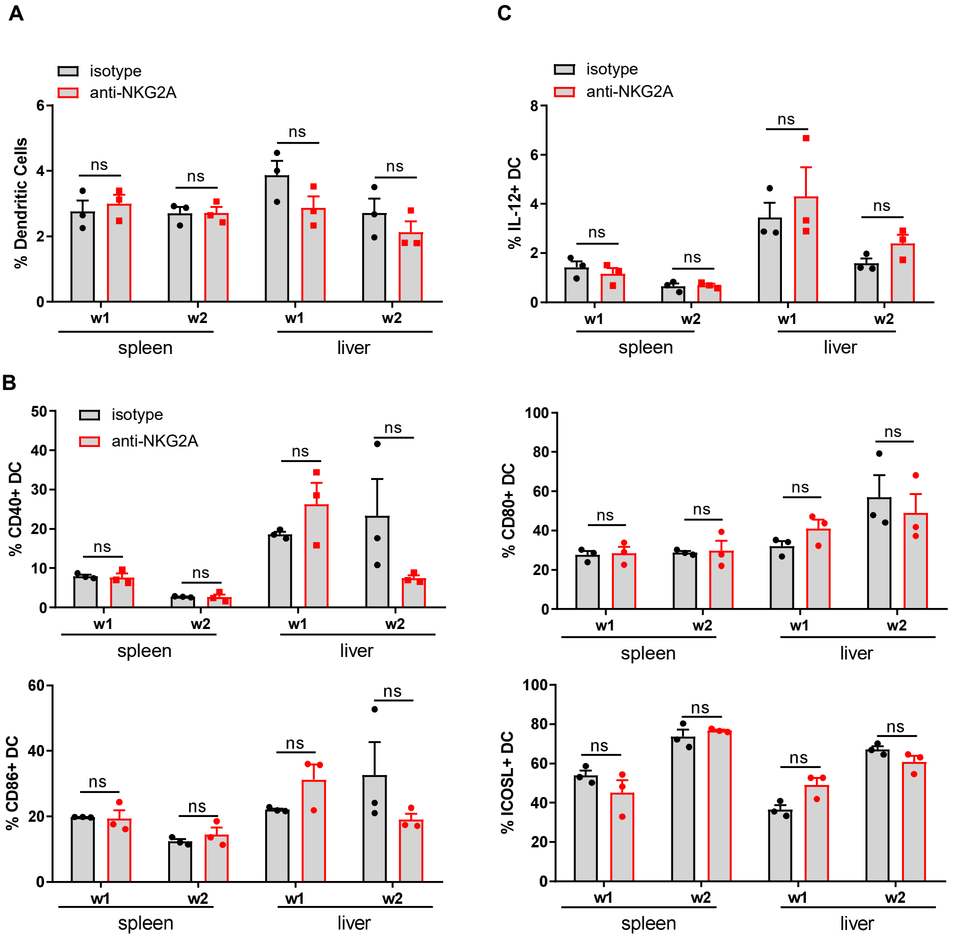

Supplementary Figure 10. Restored NK function by anti-NKG2A did not elevate T cell response via DC activation.

Mice were treated as in Figure 3D. (**A**) FACS analysis of DC percentage. (**B-C**) FACS analysis of DC cells for expression of (**B**) CD40, CD80, CD86, ICOSL and (**C**) intracellular IL-12 in spleen and liver. Data were mean ± SD, ns, not significant. Source data are provided as a Source Data file.

Supplementary Table 1. J399EM (HCV 2a) peptide information

peptide simble	HCV region	peptide sequence	H2 haplotype
**KV8**	NS3	KSIDFIPV	H-2-Kb
**YL9**	NS3	YYRGLDVSI	H-2-Kd
**QL9**	NS4B	QYLAGLSTL	H-2-Kd
**SI9**	NS5A	SEALQQLAI	H-2-Kk
**CL9**	NS5B	CEKMALYDI	H-2-Kk
**RL8**	NS5B	RSLLFGLL	H-2-Kb
**SL8**	E2	VVLLFLLL	H-2-Kb
**WL9**	E2	WEWVVLLFL	H-2-Kk
**VL8**	Core	SIFLLALL	H-2-Kb
**RA10**	Core	VNYATGNL	H-2-Kb

Supplementary Table 2. HCV 1b peptide pool information

Pool Name	Sequence	Pool Name	Sequence	Pool Name	Sequence	Pool Name	Sequence
NS3_1	LLAPITAYSQQTRGLLGC	NS4B	FDEMEECASHLPYIEQGM	NS5A_2	GSPPSLASSSASQLSAPS	NS5B_2	GQRVEFLVNAWKSKKCPM
	SQQTRGLLGCIITSLTGR		SHLPYIEQGMQLAEQFKQ		SSASQLSAPSLKATCTTH		NAWKSKKCPMGFSYDTRC
	GCIITSLTGRDKNQVEGE		GMQLAEQFKQKALGLLQT		PSLKATCTTHHDSPDADL		PMGFSYDTRCFDSTVTES
	GRDKNQVEGEVQVVSTAT		KQKALGLLQTATKQAEAA		THHDSPDADLIEANLLWR		RCFDSTVTESDIRVEESI
	GEVQVVSTATQSFLATCV		QTATKQAEAAAPVVESKW		DLIEANLLWRQEMGGNIT		ESDIRVEESIYQCCDLAP
	ATQSFLATCVNGVCWTVY		AAAPVVESKWRALETFWA		WRQEMGGNITRVESENKV		SIYQCCDLAPEARQAIRS
	CVNGVCWTVYHGAGSKTL		KWRALETFWAKHMWNFIS		ITRVESENKVVILDSFDP		APEARQAIRSLTERLYIG
	VYHGAGSKTLAGPKGPIT		WAKHMWNFISGIQYLAGL		KVVILDSFDPLRAEEDER		RSLTERLYIGGPLTNSKG
	TLAGPKGPITQMYTNVDQ		ISGIQYLAGLSTLPGNPA		DPLRAEEDEREVSVAAEI		IGGPLTNSKGQNCGYRRC
	ITQMYTNVDQDLVGWPAP		GLSTLPGNPAIASLMAFT		EREVSVAAEILRKTRKFP		KGQNCGYRRCRASGVLTT
	DQDLVGWPAPPGARSLTP		PAIASLMAFTASITSPLT		EILRKTRKFPPAMPIWAR		RCRASGVLTTSCGNTLTC
	APPGARSLTPCTCGSSDL		FTASITSPLTTQSTLLFN		FPPAMPIWARPDYNPPLL		TTSCGNTLTCYLKASAAC
	TPCTCGSSDLYLVTRHAD		LTTQSTLLFNILGGWVAA		ARPDYNPPLLESWKDPDY		TCYLKASAACRAAKLQDC
	DLYLVTRHADVIPVRRRG		FNILGGWVAAQLAPPSAA		LLESWKDPDYVPPVVHGC		ACRAAKLQDCTMLVNGDD
	ADVIPVRRRGDSRGSLLS		AAQLAPPSAASAFVGAGI		DYVPPVVHGCPLPPTKAP		DCTMLVNGDDLVVICESA
	RGDSRGSLLSPRPVSYLK		AASAFVGAGIAGAAVGSI		GCPLPPTKAPPIPPPRRK		DDLVVICESAGTQEDAAS
	LSPRPVSYLKGSSGGPLL		GIAGAAVGSIGLGKVLVD		APPIPPPRRKRTVVLTES		SAGTQEDAASLRVFTEAM
	LKGSSGGPLLCPSGHAVG	NS5A_1	STPCSGSWLRDVWDWICT		RKRTVVLTESTVSSALAE		ASLRVFTEAMTRYSAPPG
	LLCPSGHAVGIFRAAVCT		LRDVWDWICTVLTDFKTW		ESTVSSALAELATKTFGS		AMTRYSAPPGDPPQPEYD
	VGIFRAAVCTRGVAKAVD		CTVLTDFKTWLQSKLLPR		AELATKTFGSSGSSAIDS		PGDPPQPEYDLELITSCS
	CTRGVAKAVDFVPVESME		TWLQSKLLPRLPGVPFLS		GSSGSSAIDSGTATAPPD		YDLELITSCSSNVSVAHD
	VDFVPVESMETTMRSPVF		PRLPGVPFLSCQRGYKGV		DSGTATAPPDQASDDGDK		CSSNVSVAHDASGKRVYY
	METTMRSPVFTDNSSPPA		LSCQRGYKGVWRGDGIMQ		PDQASDDGDKGSDVESYS		HDASGKRVYYLTRDPTTP
	VFTDNSSPPAVPQTFQVA		GVWRGDGIMQTTCPCGAQ		DKGSDVESYSSMPPLEGE		YYLTRDPTTPLARAAWET
	PAVPQTFQVAHLHAPTGS		MQTTCPCGAQITGHVKNG		YSSMPPLEGEPGDPDLSD		TPLARAAWETARHTPVNS
	VAHLHAPTGSGKSTKVPA		AQITGHVKNGSMRIVGPR		GEPGDPDLSDGSWSTVSE		
NS3_2	GSGKSTKVPAAYAAQGYK		NGSMRIVGPRTCSNTWHG		SDGSWSTVSEEASEDVVC		
	PAAYAAQGYKVLVLNPSV		PRTCSNTWHGTFPINAYT		SEEASEDVVCCSMSYTWT		
	YKVLVLNPSVAATLGFGA		HGTFPINAYTTGPCTPSP	NS5B_3	ETARHTPVNSWLGNIIMY		
	SVAATLGFGAYMSKAHGT		YTTGPCTPSPAPNYSRAL		NSWLGNIIMYAPTLWARM		
	GAYMSKAHGTDPNIRTGV		SPAPNYSRALWRVAAEEY		MYAPTLWARMILMTHFFS		
	GTDPNIRTGVRTITTGAP		ALWRVAAEEYVEVTRVGD		RMILMTHFFSILLAQEQL		
	GVRTITTGAPITYSTYGK		EYVEVTRVGDFHYVTGMT		FSILLAQEQLEKALDCQI		
	APITYSTYGKFLADGGCS		GDFHYVTGMTTDNVKCPC		QLEKALDCQIYGACYSIE		
	GKFLADGGCSGGAYDIII		MTTDNVKCPCQVPAPEFF		QIYGACYSIEPLDLPQII		
	CSGGAYDIIICDECHSTD		PCQVPAPEFFTEVDGVRL		IEPLDLPQIIERLHGLSA		
	IICDECHSTDSTTILGIG		FFTEVDGVRLHRYAPACK		IIERLHGLSAFSLHSYSP		
	TDSTTILGIGTVLDQAET		RLHRYAPACKPLLRDEVA		SAFSLHSYSPGEINRVAS		
	IGTVLDQAETAGARLVVL		CKPLLRDEVAFQVGLNQY		SPGEINRVASCLRKLGVP		
	ETAGARLVVLATATPPGS		VAFQVGLNQYLVGSQLPC		ASCLRKLGVPPLRVWRHR		
	VLATATPPGSVTVPHPNI		QYLVGSQLPCEPEPDVAV		VPPLRVWRHRARSVRAKL		
	GSVTVPHPNIEEVALSNT		PCEPEPDVAVLTSMLTDP		HRARSVRAKLLSQGGRAA		
	NIEEVALSNTGEIPFYGK		AVLTSMLTDPSHITAETA		KLLSQGGRAATCGKYLFN		
	NTGEIPFYGKAIPIETIK		DPSHITAETAKRRLARGS		AATCGKYLFNWAVRTKLK		
	GKAIPIETIKGGRHLIFC		TAKRRLARGSPPSLASSS		FNWAVRTKLKLTPIPAAS		
	IKGGRHLIFCHSKKKCDE				LKLTPIPAASRLDLSGWF		
	FCHSKKKCDELAAKLSGL				ASRLDLSGWFVAGYSGGD		
	DELAAKLSGLGLNAVAYY				WFVAGYSGGDIYHSLSRA		
	GLGLNAVAYYRGLDVSVI				GDIYHSLSRARPRWFMLC		
	YYRGLDVSVIPTSGDVVV				RARPRWFMLCLLLLSVGV		
	VIPTSGDVVVVATDALMT				LCLLLLSVGVGIYLLPNR		
	VVVATDALMTGFTGDFDS				SVGVGIYLLPNR		
