# Supplementary material for: NKG2A is a NK cell exhaustion checkpoint for HCV persistence
Source: Nat Commun. 2019 Apr 3;10:1507. doi: 10.1038/s41467-019-09212-y (PMC6447531; doi:10.1038/s41467-019-09212-y)
Supplement: Supplementary file 3 — Reporting Summary [file 41467_2019_9212_MOESM3_ESM.pdf]

nature research

Corresponding author(s): Hong Tang

Last updated by author(s): Feb 2, 2019

Reporting Summary

Nature Research wishes to improve the reproducibility of the work that we publish. This form provides structure for consistency and transparency in reporting. For further information on Nature Research policies, see Authors & Referees and the Editorial Policy Checklist.

Statistics

For all statistical analyses, confirm that the following items are present in the figure legend, table legend, main text, or Methods section.

n/a	Confirmed	
☐	☒	The exact sample size (*n*) for each experimental group/condition, given as a discrete number and unit of measurement
☐	☒	A statement on whether measurements were taken from distinct samples or whether the same sample was measured repeatedly
☐	☒	The statistical test(s) used AND whether they are one- or two-sided<br>*Only common tests should be described solely by name; describe more complex techniques in the Methods section.*
☒	☐	A description of all covariates tested
☐	☒	A description of any assumptions or corrections, such as tests of normality and adjustment for multiple comparisons
☐	☒	A full description of the statistical parameters including central tendency (e.g. means) or other basic estimates (e.g. regression coefficient) AND variation (e.g. standard deviation) or associated estimates of uncertainty (e.g. confidence intervals)
☒	☐	For null hypothesis testing, the test statistic (e.g. *F*, *t*, *r*) with confidence intervals, effect sizes, degrees of freedom and *P* value noted<br>*Give P values as exact values whenever suitable.*
☒	☐	For Bayesian analysis, information on the choice of priors and Markov chain Monte Carlo settings
☒	☐	For hierarchical and complex designs, identification of the appropriate level for tests and full reporting of outcomes
☒	☐	Estimates of effect sizes (e.g. Cohen's *d*, Pearson's *r*), indicating how they were calculated

Our web collection on statistics for biologists contains articles on many of the points above.

Software and code

Policy information about availability of computer code

Data collection	The BD FACSDiva software version 6 was used to collect the Flow cytometry data.
Data analysis	ELISpot data were analyzed with software Immunospot 5.0.32; The flow cytometry data were analyzed with FlowJo software version 7.6; All statistical analyses were performed with GraphPad Prism software version 5.

For manuscripts utilizing custom algorithms or software that are central to the research but not yet described in published literature, software must be made available to editors/reviewers. We strongly encourage code deposition in a community repository (e.g. GitHub). See the Nature Research guidelines for submitting code & software for further information.

Data

Policy information about availability of data

All manuscripts must include a data availability statement. This statement should provide the following information, where applicable:

- Accession codes, unique identifiers, or web links for publicly available datasets
- A list of figures that have associated raw data
- A description of any restrictions on data availability

The authors declare that all data supporting the findings of this study are available within the article and its Supplementary Information files or from the corresponding authors on reasonable request.

Field-specific reporting

Please select the one below that is the best fit for your research. If you are not sure, read the appropriate sections before making your selection.

☒ Life sciences ☐ Behavioural & social sciences ☐ Ecological, evolutionary & environmental sciences

For a reference copy of the document with all sections, see nature.com/documents/nr-reporting-summary-flat.pdf

Life sciences study design

All studies must disclose on these points even when the disclosure is negative.

Sample size	The required sample size was determined before the start of the study, corresponding number and tatistical methods were shown on the figure legends.
Data exclusions	No data were excluded from the analyses in the study.
Replication	In compliance with ethical guidelines to minimize the number of animals used, we usually used a minimum of 3 mice for each data point to ensure statistical power. In vitro analyses (HCV copies, cytokines and chemokines) were usually performed on specimen (sera, liver) from animals at each time point to ensure a minimal 3 biological replicates.
Randomization	Mice are grouped with the matched age, gender, body weight and timing of experiments. To form the cohort, each batch of infection, for example, contained the number of mice more than the number of data points, to ensure the randomization and accidental exclusion of animals. The cohort was then grouped using randomly selected animals from the batches of infection.
Blinding	The investigators were not blinded to group allocation during data collection and analysis.

Reporting for specific materials, systems and methods

We require information from authors about some types of materials, experimental systems and methods used in many studies. Here, indicate whether each material, system or method listed is relevant to your study. If you are not sure if a list item applies to your research, read the appropriate section before selecting a response.

Materials & experimental systems

n/a	Involved in the study	
☐	☒	Antibodies
☐	☒	Eukaryotic cell lines
☒	☐	Palaeontology
☐	☒	Animals and other organisms
☒	☐	Human research participants
☒	☐	Clinical data

Methods

n/a	Involved in the study	
☒	☐	ChIP-seq
☐	☒	Flow cytometry
☒	☐	MRI-based neuroimaging

Antibodies

Antibodies used	Methods,"Flow cytometry" section
Validation	The details of the antibody validation are stated on the manufacturer's websites.

Eukaryotic cell lines

Policy information about cell lines

Cell line source(s)	Methods,"NK Cell-Mediated Cytotoxicity Assay" section.
Authentication	Cell lines were authenticated by the providers.
Mycoplasma contamination	Cells were treated with mycoplasma removal reagents before using.
Commonly misidentified lines (See ICLAC register)	N/A

Animals and other organisms

Policy information about studies involving animals; ARRIVE guidelines recommended for reporting animal research

Laboratory animals	Methods,"Mice" section.
Wild animals	N/A
Field-collected samples	The study did not involve samples collected from the field.
Ethics oversight	The study received ethical approval from Institute of Biophysics, Chinese Academy of Sciences Institutional Laboratory Animal Care and Use Committee.

Note that full information on the approval of the study protocol must also be provided in the manuscript.

Flow Cytometry

Plots

Confirm that:

☒ The axis labels state the marker and fluorochrome used (e.g. CD4-FITC).

☒ The axis scales are clearly visible. Include numbers along axes only for bottom left plot of group (a 'group' is an analysis of identical markers).

☒ All plots are contour plots with outliers or pseudocolor plots.

☒ A numerical value for number of cells or percentage (with statistics) is provided.

Methodology

Sample preparation	Methods, "Flow cytometry" section.
Instrument	BD LSRFortessa
Software	The experiment data were collected by Diva software version 6 (BD Biosciences, USA) and analyzed by FlowJo software version 7.6 (Tree StarOR).
Cell population abundance	The abundance of relevant cell populations were described on relevant places.
Gating strategy	The gating strategies were shown in Supplementary Figure 3.

☒ Tick this box to confirm that a figure exemplifying the gating strategy is provided in the Supplementary Information.
